# Supplementary material for: The infraslow fluctuation of sigma power during sleep and its links to markers of arousal and memory reactivation across development
Source: Sci Rep. 2026 Jun 18;16:27766. doi: 10.1038/s41598-026-58423-z (PMC13542187; doi:10.1038/s41598-026-58423-z)
Supplement: Supplementary file 1 [file 41598_2026_58423_MOESM1_ESM.docx]

**Supplementary Table 1**: Electrophysiological and Sleep Bout Characteristics for the Temporal Dynamics of Sigma Power Time Course Analyses

| **Parameters** | **CH** | **EA** | **LA** | **YA** |  |  |
| --- | --- | --- | --- | --- | --- | --- |
|  | Mean (SD) | Mean (SD) | Mean (SD) | Mean (SD) | p-value | Corrected p-value and Direction |
|  |  |  |  |  |  |  |
| **Electrophysiological Characteristics** | |  |  |  |  |  |
| Sleep Spindles (#) | 721.26 (288.44) | 727.91 (294.66) | 826.25 (292.16) | 812.24 (337.09) | 0.32 |  |
| Sleep Spindle Density (# per min) | 4.69 (0.74) | 4.84 (0.93) | 5.05 (0.96) | 5.00 (1.08) | 0.22 |  |
| Microarousals (#) | 9.97 (9.25) | 12.31 (18.25) | 10.41 (7.05) | 9.63 (6.24) | 0.77 |  |
| Microarousal Density (# per hour) | 4.53 (6.02) | 4.97 (7.07) | 3.86 (2.35) | 3.50 (1.91) | 0.74 |  |
| Microarousal Duration (s) | 7.63 (2.08) | 8.10 (2.16) | 7.51 (2.21) | 7.95 (2.11) | 0.72 |  |
| High-Amplitude Slow Waves (#) | 844.18 (372.02) | 787.22 (317.04) | 883.84 (342.78) | 913.32 (362.43) | 0.52 |  |
| High-Amplitude Slow Wave Density (# per min) | 5.47 (1.06) | 5.31 (1.28) | 5.40 (1.26) | 5.63 (0.98) | 0.83 |  |
| High-Amplitude Slow Wave-Sleep Spindle Couplings (#) | 74.00 (32.33) | 73.51 (35.56) | 96.94 (54.28) | 99.24 (50.70) | **0.04** | n.s. |
| High-Amplitude Slow Wave-Sleep Spindle Coupling Density (# per min) | 0.48 (0.11) | 0.49 (0.18) | 0.58 (0.24) | 0.60 (0.19) | **0.01** | CH<YA (**0.02**) EA<YA (**0.02**) |
|  |  |  |  |  |  |  |
| **Sleep Bout Characteristics** |  |  |  |  |  |  |
| Bouts (#) | 14.10 (4.17) | 14.69 (3.68) | 15.53 (3.32) | 14.21 (2.84) | 0.57 |  |
| Mean Bout Length (s) | 641.86 (158.85) | 596.08 (102.35) | 623.42 (115.18) | 682.33 (194.12) | 0.31 |  |
| Total Duration of Bouts (min) | 152.91 (58.03) | 147.08 (45.71) | 161.10 (41.59) | 160.06 (47.08) | 0.55 |  |
| Mean Relative Location (%) | 53.78 (6.85) | 54.61 (7.46) | 53.52 (5.93) | 51.28 (5.81) | 0.11 |  |
| Proportion of all N2 | 69.88 (15.89) | 67.94 (13.81) | 74.09 (11.04) | 70.49 (15.26) | 0.33 |  |

**Supplementary Table 1:** CH: Children; EA: Early adolescents; LA: Late adolescents; YA: Young adults; SD: Standard Deviation; N2: Non-Rapid Eye Movement Stage N2; #: number; min: minutes; s: seconds; %: percent; n.s.: non-significant following Tukey-Kramer post-hoc tests. Electrophysiological characteristics and bout sleep parameters used for the temporal dynamics of the sigma power time course analyses. Electrophysiological characteristics include the sleep spindle number and density (number per minute), microarousal number, density (number per hour), and duration (in seconds), high-amplitude slow wave number and density (number per minute), and high-amplitude slow wave-sleep spindle coupling number and density (number per minute). Bout: N2 sleep data that lasts for at least 280 seconds and is uninterrupted by artifacts or transitions to other vigilance states. Mean Relative Location: the mean across all bout midpoints divided by the total length of the recording. Proportion of all N2: the total duration of bout data is divided by the total amount of N2 sleep. Corrected p-values and the direction of the difference between age groups is plotted with significantly different p-values written in bold.

**Supplementary Table 2**: Distribution of Electrophysiological Characteristics Across Phases of the Infraslow Fluctuation of Sigma Power

| **Electrophysiological Characteristics** | **ISFS Positive Half-Wave** | **ISFS Negative Half-Wave** | **Not ISFS** |
| --- | --- | --- | --- |
| Sleep Spindles | 84,508/118,190 (71.5%) | 23,760/118,190 (20.1%) | 9,922/118,190 (8.4%) |
| Microarousals | 1,154/1,642 (70.3%) | 314/1,642 (19.1%) | 174/1,642 (10.6%) |
| High-Amplitude Slow Waves | 74,938/131,337 (57.1%) | 43,330/131,337 (33.0%) | 13,069/131,337 (9.9%) |
| High-Amplitude Slow Waves Coupled to Sleep Spindles | 9,561/13,067 (73.2%) | 2,417/13,067 (18.5%) | 1,089/13,067 (8.3%) |

**Supplementary Table 2:** The table shows the distribution of electrophysiological events across the positive and negative half-waves of the infraslow fluctuation of sigma power (ISFS), as well as during periods outside the ISFS. For each event type, both the absolute number of occurrences and the percentage of the total are reported (pooled across all age groups). As expected, all electrophysiological characteristics were most likely to occur during the positive half-wave, less likely during the negative half-wave, and least likely outside the ISFS.

**Supplementary Figure 1**: Examples of Microarousals in the Presence and Absence of Sleep Spindles

**Supplementary Figure 1**: EEG: electroencephalography; EMG: electromyography. Examples of microarousals (A) with sleep spindles and (B) without sleep spindles. Detected sleep spindles are highlighted in blue in the EEG trace (top row) and EMG trace is shown (bottom row). The onset and offset of the detected microarousals are indicated by dashed pink vertical lines.

**Supplementary Figure 2**: Distribution of Microarousals within the Infraslow Fluctuation of Sigma Power Using Different Inclusion Thresholds

**Supplementary Figure 2: A)** The distribution of the onset of microarousals across bins of the infraslow fluctuation of sigma power (ISFS) and in periods where the ISFS could not be estimated (denoted as "Not ISFS") is displayed. Participants are excluded from this analysis if they did not have at least one microarousal. (**B)** The distribution of the onset of microarousals across bins of the ISFS and in the Not ISFS is displayed. Participants are excluded from this analysis if they did not have at least ten microarousals. All microarousals are calculated by dividing the number of microarousals in each phase bin of the ISFS by the total number of microarousals for each participant, with the mean across participants displayed. Each column represents an age group. For groups and parameters with many significant differences between groups, grey arrows are used to show groups of phase bins that are significantly higher when compared to other phase bins (denoted in grey brackets); the range of p-values written above them in grey. The bold black wave illustrates the relative position of each phase bin within the ISFS.

**Supplementary Figure 3:** Distribution of Beta Microarousals and 1.5+ Second Microarousals within the Infraslow Fluctuation of Sigma Power

**Supplementary Figure 3: A)** The distribution of the onset of beta microarousals across bins of the infraslow fluctuation of sigma power (ISFS) and in periods where the ISFS could not be estimated (denoted as "Not ISFS") is displayed. (**B)** The distribution of the onset of 1.5+ second microarousals (i.e., using a less rigorous minimum length criterion to potentially include shorter microarousals present in child and adolescent populations) across bins of the ISFS and in the Not ISFS is displayed. Participants are excluded from this analysis if they did not have at least one microarousal. All microarousals are calculated by dividing the number of microarousals in each phase bin of the ISFS by the total number of microarousals for each participant, with the mean across participants displayed. Each column represents an age group, with significant differences between phase bins marked by asterisk(s). For groups and parameters with many significant differences between groups, grey arrows are used to show groups of phase bins that are significantly higher when compared to other phase bins (denoted in grey brackets); the range of p-values written above them in grey. The bold black wave illustrates the relative position of each phase bin within the ISFS.
